# Pain following Craniotomy: Reassessment of the Available Options

**DOI:** 10.1155/2015/509164

**Published:** 2015-10-01

**Authors:** Rudrashish Haldar, Ashutosh Kaushal, Devendra Gupta, Shashi Srivastava, Prabhat K. Singh

**Affiliations:** Department of Anaesthesiology, Sanjay Gandhi Post Graduate Institute of Medical Sciences, Rae Bareilly Road, Lucknow, Uttar Pradesh 226014, India

## Abstract

Pain following craniotomy has frequently been neglected because of the notion that postcraniotomy patients do not experience severe pain. However a gradual change in this outlook is observed because of increased sensitivity of neuroanaesthesiologists and neurosurgeons toward acute postcraniotomy pain. Multiple modalities exist for treating this variety of pain each with its own share of advantages and disadvantages. However, individually none of these modalities has been proclaimed as the best and applicable universally. A considerable amount of dispute remains to ascertain the appropriate therapeutic regimen for treating postcraniotomy pain in spite of numerous trials using different drugs and their combinations. This review aims to highlight the genesis, characteristics, and different strategies that are undertaken for management of acute postcraniotomy pain. Chronic postcraniotomy pain which can be debilitating sequelae is also discussed concisely.

## 1. Introduction

Postcraniotomy patients are often assumed to experience lower degree of pain. Suggested reasons include lesser number of pain receptors in dura, pain insensitivity of the brain, reduced pain fibre density along the incision lines, or development of autoanalgesia [1]. Hence postcraniotomy pain has often being overlooked and traditionally has been a subject of inconsistent research. The widely held belief that craniotomy pain is modest is currently a debatable issue in face of gradually accumulating evidence [2, 3]. Approximately 60% of the patients experience moderate to severe pain [2] and its veracity has been established by several prospective studies [3–6]. As a result of inadequate analgesic therapies, patients continue to endure pain (often severe) especially in the first-postoperative hour which might extend up to first- or second-postoperative day [7, 8]. Not only is unsatisfactory pain relief distressing for the patient, it also forms the basis of various postoperative complications and prolonged hospital stay and increases healthcare expenditures [8]. From the neurosurgical perspective, pain associated sympathetic stimulation leads to hypertension which has the inherent potential of precipitating secondary intracranial haemorrhage [9]. On the other hand, overzealous attempts at pain control may be accompanied by excessive sedation which camouflages the new onset neurological deficits and hamper the neurological response monitoring. Depressed respiration can give rise to hypercarbia which increases cerebral blood volume and consequently raise the intracranial pressures (ICP). Thus in face of these conflicting scenarios, perioperative caregivers often undertake excessively conservative approach for pain relief. Therefore postoperative pain following craniotomy remains an area where conventional pain management strategies often fail to meet their objectives. In the absence of solid evidence based guidelines, administration of appropriate postoperative analgesia in postcraniotomy cases is difficult [10]. A limited number of evidence based studies often generating contradictory results have led to use of inconsistent therapeutic measures leading to suboptimal care. Thus the potential for exploring the “gold standard” regimen for postcraniotomy pain relief still exists. This review attempts to explore the relevant literature and highlight the various therapeutic options available for acute postcraniotomy pain relief. A concise overview of the development of chronic postcraniotomy pain, the pathophysiology of chronicity, and remedial measures is also attempted in the later part of the review.

## 2. Characteristics of Acute Pain following Craniotomy

Postcraniotomy pain is usually pulsating or pounding in nature similar to tension headaches. Sometimes it can be steady and continuous. Postcraniotomy pain normally afflicts women and young patients [11, 12]. The pain is a consequence of surgical incision and reflection of pericranial muscles and soft tissues of the scalp and thus has somatic origins. Suboccipital and subtemporal approaches involving considerable dissection of major muscles like temporal, splenius capitis, and cervicis are associated with the highest incidence of pain [13]. Skull base surgeries employing these approaches produce higher degree of postoperative pain [14]. Dunbar et al. however observed that patients who had undergone frontal craniotomy reported higher pain scores [1]. Meningeal irritation also contributes to postsurgical pain. Nevertheless it is the amount of tissue damage rather than the location of the surgery, which determines the intensity of postcraniotomy pain [10]. Greater amount of tissue injury generates higher intensity of postoperative pain. Postsurgical cerebrospinal fluid (CSF) leakage can occur following skull base surgeries which can be responsible for headaches. Headaches due to CSF leaks show considerable variability. In majority of the times it is orthostatic in nature. Even if it is lingering or steady, it is aggravated during upright position and decreases with recumbency [15].

## 3. Classification and Assessment of Postcraniotomy Pain

The International Classification of Headache Disorders (ICHD-3) published by the International Headache Society which lays down diagnostic criteria for different headaches has classified postcraniotomy headache and subdivided into acute and persistent varieties. The descriptions of the varieties are as follows.

### 3.1. Acute Headache Attributed to Craniotomy

*Description*. Headache is of less than 3 months' duration caused by surgical craniotomy.

*Diagnostic Criteria.* They are as follows:

- Any headache fulfilling criteria (C) and (D).
- Surgical craniotomy which has been performed.
- Headache which is reported to have developed within 7 days after one of the following:
  1. the craniotomy,
  2. regaining of consciousness following the craniotomy,
  3. discontinuation of medications that impair ability to sense or report headache following the craniotomy.
- Either of the following:
  1. headache resolved within 3 months after the craniotomy,
  2. headache not yet resolved but 3 months have not yet passed since the craniotomy.
- Not better accounted for by another ICHD-3 diagnosis.

### 3.2. Persistent Headache Attributed to Craniotomy

*Description*. Headache is of greater than 3 months' duration caused by surgical craniotomy. 

*Diagnostic Criteria.* They are as follows:

- Any headache fulfilling criteria (C) and (D).
- Surgical craniotomy which has been performed.
- Headache which is reported to have developed within 7 days after one of the following:
  1. the craniotomy,
  2. regaining of consciousness following the craniotomy,
  3. discontinuation of medication that impairs ability to sense or report headache following the craniotomy.
- Headache persisting for >3 months after the craniotomy.
- Not better accounted for by another ICHD-3 diagnosis [16].

Exact quantification of pain in postcraniotomy patients is problematic as the patients should be capable of perceiving and expressing pain which might not be always possible following neurosurgical procedures. Subjective assessments by observing acute pain behavior may be required. However alert and oriented patients can be asked to rate their pain numerically (1–10) [1] or using visual analogue scale (VAS) [17].

## 4. Therapeutic Measures to Manage Acute Postcraniotomy Pain

Postcraniotomy pain management is an unorganised sphere owing to the dearth of standard analgesic protocols. Besides the intraoperative anaesthetic techniques or opioid usage may be variable in different patients or surgical settings which have a bearing on the postoperative pain. Moreover, altered neurological status following neurosurgical procedures and the subjective nature of pain assessment hampers the appropriate quantification of pain. Perioperative clinicians often restrict prescribing analgesics (especially opioids) in apprehension of their potential side effects and variable regimens are followed. Hence as of now, we lack a consensus on the ideal line of management of postcraniotomy pain.

## 5. Local Anaesthetics

- Scalp block: scalp block includes infiltrating local anaesthetics to seven nerves on either side of the scalp. The nerves and a brief description of the technique are as follows [18, 19]:
- After cleaning the skin with chlorhexidine or Betadine, the following nerves are blocked on the scalp by infiltrating local anaesthetics using a 23-gauge needle:
  - supraorbital nerve (a branch of Trigeminal Nerve): the supraorbital notch is palpated, needle is inserted perpendicularly at the upper orbital margin, and 1–3 mL of the drug is injected,
  - supratrochlear nerve (a branch of Trigeminal Nerve): the needle is inserted medial to the point of supraorbital nerve injection site and 1–3 mL of the drug is spread medially while injecting above the eyebrow line,
  - zygomaticotemporal nerve (a branch of Trigeminal Nerve): local anaesthetic needs to be infiltrated superficial and deep to the temporalis muscle. The needle is inserted at the lateral edge of the supraorbital margin (1 cm lateral and 1 cm superior to the lateral canthus of the eye) where 3–5 mL of drug infiltration is done and continues to the distal aspect of the zygomatic arch,
  - auriculotemporal nerve (a branch of Trigeminal Nerve): local anaesthetic is infiltrated 1 cm anterior to the auricle above the level of the temporomandibular joint. Superficial temporal artery should be palpated beforehand to avoid intra-arterial injection,
  - greater auricular nerve (a branch of second- and third-cervical spinal nerve): infiltration of 3–5 mL local anaesthetic is done subcutaneously at the level of tragus, behind the auricle,
  - lesser occipital nerve (a branch of the second- or third-cervical spinal nerve): infiltrate 3–5 mL of local anaesthetic subcutaneously behind the auricle starting from the top-down to the auricular lobule and then continue to infiltrate along the superior nuchal line to thegreater occipital nerve,
  - greater occipital nerve (a branch of the second-cervical spinal nerve): after palpating the occipital artery, which is found about 3-4 cm lateral to the external occipital protuberance along the superior nuchal line, 3–5 mL local anaesthetic is injected medial to the occipital artery between mastoid process and occipital protuberance.
- The prominent benefit offered by scalp block is the ability to perform accurate neurological assessment postoperatively as it does not affect other motor or sensory modalities. Scalp block has shown to decrease the frequency of request for rescue analgesics, increase the time between completion of surgery and first request of analgesics, and decrease pain scores in the initial postoperative phase [20]. Ropivacaine (0.75%) scalp block has been seen to decrease postcraniotomy pain [21]. Scalp block also facilitates “transitional analgesia” following remifentanyl based intraoperative analgesia [22]. The scalp is richly innervated by C fibres and ropivacaine having selective action on the sensory A*δ* and C fibres is a favourable agent.
- Infiltration of wound margins: preincisional infiltration of local anaesthetics produces negligible effect on postoperative pain following craniotomies. However infiltrating the wound margins can cause a modest decrease in the pain intensity [23]. This outcome is vital especially in reducing the development of chronic pain irrespective of its inflammatory or neuropathic basis. Bupivacaine infiltration (0.25% with adrenaline) before surgery and following skin closure has shown to decrease postoperative pain scores up to one hour following surgery [24].

Possibly local anaesthetics exert their effects through preemptive analgesic mechanisms [21]. However the major limitation of this modality is that the duration of pain relief is limited to the initial few hours following surgery. As the effect of local anaesthetics subside, additional pharmacotherapy is needed to control pain. Inability to repeat local anaesthetic injections following sterile dressing is another drawback. Vigilance has to be maintained to avoid local anaesthetic toxicity as rapid rises in serum local anaesthetic concentration can occur. Haematoma, infections, and intra-arterial or subarachnoid injections can be rare complications of scalp blocks. Amongst the two techniques, wound infiltration with local anaesthetics appears to have a favorable pain control profile than scalp block [25].

## 6. Opioids

In spite of the controversies surrounding their use in the neurosurgical population, opioids form the mainstay of management of moderate to severe pain. Commonly used opioids for postcraniotomy analgesia include morphine, codeine, fentanyl, and tramadol. Their action is mediated via specific opioid receptors in the central and peripheral nervous system. Concerns related to respiratory depression, sedation, hypercarbia, increased ICP, and delayed weaning from ventilator exist with opioid therapy. Concerns regarding their addictive potential or them being the last resort treatment are also present. These traditional conceptions have limited the widespread use of opioids following neurosurgery thereby compromising the adequacy of analgesia. However, systemic opioids are frequently needed for providing adequate pain relief following craniotomies. Opioid administration can be either parenteral or enteral.

### 6.1. Parenteral

(a) Morphine: parenteral morphine can be administered through intravenous (including PCA) or intramuscular routes. The potent analgesic effects blunt the haemodynamic surges during recovery from anaesthesia or in the early postoperative period safeguarding against possible intracranial hemorrhage. PCA facilitates the patients to control pain by themselves and also decreases the overall opioid consumption. Reduction in pain scores, higher patient satisfaction, and absence of side effects (with coadministered antiemetics and vigilant monitoring) are the advantages achieved with this mode of analgesia [26]. However the need for an intact sensorium and alertness are the limitations which precludes the extensive application of PCA devices.
 Administering intramuscular injections of morphine is also an accepted practice although the drawbacks include a slower onset, variable systemic absorption, and pain at the injection site.
(b) Fentanyl: compared to morphine, it is more potent, lipophilic, and faster acting. Because of its shorter duration of action it is prudent to administer this drug via PCA; nevertheless it can be used intravenously for breakthrough pain. Previous trials have demonstrated that pain control is superior when fentanyl has been used through PCA either alone [27] or in conjunction with NSAIDs [28]. Increased lucidity and patients' comfort are the additional advantages. Though transdermal fentanyl application is an upcoming and novel approach, the transdermal route is contraindicated for acute pain relief because of its delay in onset, difficulty in drug delivery, and prolonged elimination half-life. Additionally the safety of transdermal route is questionable in neurosurgical patients as subcutaneous absorption of fentanyl continues to occur for a substantial period of time following patch removal [29].
(c) Tramadol: it is a synthetic analgesic which provides analgesia via opioid mechanisms (*μ* receptor agonism) as well as nonopioid mechanisms (increasing central neuronal synaptic levels of serotonin and noradrenaline). Though the analgesic efficacy is 10–15 times lesser as compared to morphine, the side effects are relatively less. Repeated administration does not lead to dependence, ceiling effect is absent, and respiratory depression is rare. Addition of tramadol along with other narcotics in the postoperative analgesia regimen has shown to reduce postoperative pain, decrease side effects of other opioids, decrease length of stay, and reduce overall hospitalization costs [30]. Tramadol is favoured in patients with unstable cardiovascular or respiratory status. However due to the probability of distressing nausea and vomiting [31] and rare incidence of seizures [32] following bolus administration, its use merits caution.

### 6.2. Enteral

Codeine and oxycodone are the opioids usually used through enteral route especially when converting from injectable to oral therapy. The analgesic and respiratory depressant properties of these drugs are similar to morphine in equipotent doses. Ceiling effects to respiratory depression and noninterference with pupillary signs make codeine an attractive choice even though incidence of vomiting is high with codeine use [29]. Codeine is a moderately potent narcotic, which requires demethylation to active metabolite (morphine). Codeine metabolism is dependant on cytochrome P450 enzyme system (specifically CYP 2D6). Phenotypic variations in patients differentiate them into poor metabolizers (inadequate conversion to morphine resulting in poor analgesia) or ultra metabolizers (large amount of morphine production). Therefore subject to interindividual variability in biotransformation to its active metabolite, production rate and the plasma concentration of metabolites and consequently the efficacy of prodrug vary. Consequently, analgesic efficacy can be variable and insufficient. On the other hand impaired sensorium in ultra metabolizers (due to morphine overload) could be misattributed to neurological or other causes. Moreover drugs which the neurosurgical patient is taking simultaneously can inhibit CYP 2D6 and codeine metabolism [33]. To obtain synergistic potentiation, codeine and oxycodone are often coadministered with acetaminophen and aspirin. Sustained released tablets of oxycodone are unsuitable for administration through nasogastric tube as crushing the tablets releases a large amount of oxycodone for systemic uptake. On the other hand, in patients with rapid gastrointestinal transit, sustained release preparations may not be absorbed at all.

## 7. Nonopioid Analgesics

- Paracetamol: though the exact mechanism of action of paracetamol is unclear, presumed mechanisms include central antinociceptive action, inhibition of prostaglandin H_2_ synthetase, stimulating activity of descending serotoninergic pathway in spinal cord, or modulation of *β* endorphin receptors. Paracetamol is used in certain centres for postcraniotomy pain relief although as a sole agent it is ineffective for adequate pain control. However its use in conjunction with opioids and other NSAIDs reduces pain scores considerably [34, 35]. A reduction in opioid consumption has been demonstrated when paracetamol has been coadministered with PCA opioids although no change in the side effect profile has been observed [36]. Cautious use is advocated as overdosing might cause hepatotoxicity.
- NSAIDs: the use of NSAIDs in neurosurgery is a contentious issue. NSAIDS mediate their analgesic effects via inhibition of prostaglandins thereby decreasing pain and inflammation. Use of diclofenac has been advocated in the absence of bleeding disorders or renal defects [13]. Since NSAIDs inhibit platelet aggregation thereby increasing the bleeding time, the risk of postoperative bleeding persists. Moreover in the postoperative period, hypovolemia or vasoconstrictor therapy might follow craniotomy. Here the renal blood flow becomes “prostaglandin dependant.” NSAIDs can prove detrimental in such situations. Indomethacin has been demonstrated to decrease cerebral blood flow by vasoconstriction [37]. Care should be exercised during their use in the early postoperative period and vigilance is required so that haematoma formation due to deranged coagulation does not occur.
- COX-2 inhibitors: a good deal of enthusiasm was generated following the advent of selective COX 2 inhibitors considering that they acted specifically on inflammatory mediators and avoided platelet dysfunctions. Intravenous parecoxib was administered along with morphine and scalp blocks to diminish postcraniotomy pain. However the results of these trials have demonstrated insignificant differences in analgesia [38, 39]. Addition of rofecoxib reduced the oral requirement of oxycodone and simultaneously reduced the opioid related side effects and provided better analgesia [40]. Even though they reduce the narcotic consumption, decrease the duration of hospital stay, and enhanced patients satisfaction, their recommendation for routine use is nowadays debatable [30]. With the controversies surrounding the potential cardiovascular effects and thromboembolic events of this class of drugs, the initial interest in this drug class has gradually faded [41].

## 8. NMDA Receptor Antagonist

NMDA receptors are ligand gated ion channel which allow the passage of calcium, sodium, and potassium into the cell. They are involved in pain modulation at the level of spinal cord and sensitization of nociceptors. NMDA receptor antagonists lack intrinsic analgesic properties, however their analgesic effects are mediated via inhibiting central sensitization. A previous review [42] has shown a reduction in postoperative pain and analgesic requirement using dextromethorphan and ketamine. Employing ketamine in postcraniotomy patients seems injudicious considering the undesirable ICP raise, but dextromethorphan may prove to be an important constituent in the multimodal analgesia regimens following craniotomy [43].

## 9. Gabapentin

This is a new generation antiepileptic which possesses antinociceptive or antihyperalgesic properties. Investigation carried out by Türe et al. [44] has shown that preoperative administration of gabapentin has a favourable postoperative outcome in the form of reduced pain scores, lower opioid consumption, and lower incidence of nausea and vomiting. However on the flipside higher levels of sedation and delayed tracheal extubation had been the associated complications.

## 10. *α*2 Adrenoreceptor Agonist

They are the relatively new entrants in the field of pain management. Dexmedetomidine is a potent presynaptic *α*2 adrenoreceptor antagonists which provides sedation without affecting respiration. Investigations involving dexmedetomidine claim a reduction of postoperative opioid consumption by as much as 60% in intra-abdominal and orthopaedic procedures [45]. Preemptive analgesic activity of this drug has also been postulated [46]. However delayed recovery and longer discharge times from the postanaesthesia care unit (PACU) have been observed in patients receiving perioperative dexmedetomidine infusions [47]. Another utility of this class of drugs is to provide transitional analgesia between surgical anaesthesia and postanaesthesia care units.

## 11. Chronic Pain following Craniotomy

Persistent pain after suboccipital craniotomy is debilitating conditions which impairs the professional and social life of the subject. Various causes attributed to development of chronicity include dural traction [48, 49], cervical muscle destruction [49], nerve entrapment [50], or cerebrospinal fluid leakage [51]. Chronic pain may also result from uneventful supratentorial craniotomies affecting a sizeable number of patients [52]. Clinically persistent headache after craniotomy is characterized as a combination of tension type and “site of injury” headache overlying the surgical site. It can be sharp aching, pressurising, or throbbing. The surgical technique too seems to have a bearing on the postoperative pain. In the retrosigmoid approach replacement of bone flap or direct dural closure leads to higher incidence of pain [53]. Application of fibrin glue or drilling possibly leading to aseptic meningitis can be the genesis chronic pain [54, 55]. Postcraniotomy headache can also occur following scar tissue formation which involves the occipital nerves or development of fibrous adhesions which bind neck muscles to the dura. Neck movement causes traction on the dura and leads to generation of pain [56]. Chronic headache is a common aftermath following head injury afflicting a sizeable proportion of patients [57]. Such patients, following surgery for the primary head injury, have high propensity to develop chronic posttraumatic headaches.

Chronic postcraniotomy pain can be treated using nonpharmacological (TENS, acupuncture, radiofrequency or cryoablation, physiotherapy, etc.) or pharmacological therapies. Combination of the two therapies can also be tried to obtain favorable outcomes. The common medications prescribed are NSAIDs, paracetamol, or narcotics (codeine, hydrocodone, and oxycodone) [58]. Local anaesthetics in the form of trigger point injections or topical gels and patches are viable alternatives in selected cases. Along with the routine analgesics, combination therapy with newer antiepileptics like gabapentin, lamotrigine [59], topiramate, and tiagabine has been tried. In neuropathic pain associated with allodynia and hyperalgesia, gabapentin has shown promising results. Other anticonvulsants like sodium valproate is effective in migraine-like headaches associated with craniotomy of posthead trauma [60, 61]. Newer drugs like sumatriptan (5HT1 agonist) have been found useful in patients with persistent headache following acoustic neuroma excision [62].

## 12. Newer Prospects for Treating Postcraniotomy Pain

Electromyography provides a noninvasive means to detect muscular imbalance in patients following craniotomy [63]. Application of this technique can help in titrating the pharmacological management according to the individual patients. Cryotherapy has emerged as an attractive option whereby application of ice packs on wounds and periorbital areas had significantly altered pain intensity in postcraniotomy subjects [64]. Voltage gated sodium channels especially the tetrodotoxin- (TTX-) resistant channels (NaV1.8) are implicated in the development of various chronic pain syndromes. Development of drugs specifically targeting the functions of these channels will aid immensely in providing relief from chronic postcraniotomy pain.

## 13. Conclusion

Over the past few years, there has been a growing awareness and sensitivity amongst the neuroanaesthesiologists and neurosurgeons towards the necessity of providing superior quality of postoperative pain relief to the patients who have undergone craniotomy. This has translated into better pain management practices and strategies. A fundamental requirement in this class of patients is a relatively clear level of consciousness for neurological evaluation. Consequently simultaneous maintenance of appropriate neurological objectives and adequate analgesia is a delicate balancing job. Since a sizeable number of therapeutic options in addition to opioids exist, multimodal analgesia offers the rational promise of superior quality of analgesia with minimal side effects of the individual drugs. Though there is a growing volume of literature on the various modalities of treating acute pain following craniotomy, absence of consensus or uniformity in the analgesic protocol has still left this issue as a grey zone. Majority of the patients following craniotomy receive antiseizure drugs concurrently. The influence of these drugs on analgesic needs is not apparent. Thus the ideal pain management protocol along with uniform pain management practices following craniotomy still remains elusive.

Nonetheless the observations made by the various trials can be translated into realistic therapeutic approaches for treating postcraniotomy pain. A multimodal or “balanced” approach is advocated where smaller doses of opioids, NSAIDS, local anesthetics, N-methyl-D-aspartate antagonists, and *α*2-adrenergic agonists are combined to maximize pain control and minimize side effects. Application of preemptive approaches can reduce the postoperative pharmacological burden on the patients. A viable option consists of the following regimen: an appropriate block for the anticipated craniotomy can be placed before the head is secured with pins. Intraoperatively, a balanced opioid (fentanyl, remifentanyl) based technique can be utilized. Low dose intravenous NMDA antagonists have been suggested intraoperatively and should be stopped 40 minutes before the end of the procedure. In case the maximum allowable dose of local anaesthetics has not been exceeded, a scalp block or infiltration can be repeated before extubation. With the use of intraoperative remifentanil, postoperative hyperalgesia and provision of adequate transitional analgesia are additional concerns which should be controlled with long acting opioids like morphine. Pain in the postoperative period should be assessed using objective and validated methods. Opioids and intravenous paracetamol can be the first-line drugs for immediate postoperative pain relief. Though intravenous fentanyl boluses have good potency, it is limited by its short duration of action. Morphine provides longer and consistent analgesia and with careful titration and cautious monitoring, serious side effects can be avoided [65]. NSAIDs (if no contraindications), oxycodone, and tramadol can be used additionally. The use of codeine and intramuscular drug therapy should be discouraged [65]. The patients can be shifted to oral analgesics once they become conscious with intact reflexes and tolerate oral feeding. Antiemetics, laxatives, and gastric ulcer protective drugs should be coprescribed in consideration of the side effects of opioids and NSAIDs.

The need for conducting further well designed, high quality randomized control trials remains in order to establish the ideal combination therapies amongst the hosts of available options along with their dosages and regimens. This would help in making substantial progress in providing better and patient specific analgesic therapy to this subset of patients. Early and aggressive relief from pain is also imperative to prevent the transition of acute pain to chronic pain.
